# Traditional Gender Differences Create Gaps in the Effect of COVID-19 on Psychological Distress of Japanese Workers

**DOI:** 10.3390/ijerph18168656

**Published:** 2021-08-16

**Authors:** Tomoyuki Kobayashi, Masaharu Maeda, Yui Takebayashi, Hideki Sato

**Affiliations:** Department of Disaster Psychiatry, Fukushima Medical University School of Medicine, Fukushima 960-1295, Japan; masagen@fmu.ac.jp (M.M.); takeb-ky@fmu.ac.jp (Y.T.); hidekis@fmu.ac.jp (H.S.)

**Keywords:** mental health, social interaction, item response theory, gendered vulnerability, health care work

## Abstract

The COVID-19 pandemic has caused Japanese workers psychological distress through crises of health, economics, and social relationships. To assess whether these effects are amplified by the gender bias that exists in Japan, we examined male and female worker’s psychological distress and difficulties during the pandemic. An online “COVID-19-related difficulties” questionnaire, based on item response theory, gathered responses from 3464 workers in October and November 2020. The workers’ psychological distress was found concerned to be significantly worse than before the pandemic. Basic stressors related to infection anxiety, economic anxiety, and restrictions on social interactions and outings. Men’s and women’s experiences of difficulties were consistent with traditional gender roles in Japan: men were more likely to face job-related stressors, such as economic insecurity and work-style changes; women were more likely to face non-job-related stressors, such as increased living costs and reduced social interactions. Policymakers and employers should consider the association between gender differences and industry types, and implement measures to strengthen the acceptability of mental health care.

## 1. Introduction

Coronavirus disease (COVID-19) is an infectious disease that typically causes respiratory illness. The World Health Organization (WHO) first characterized the disease as a pandemic more than one year ago [1]. Although effective vaccines have been developed, it will take time for a large number people to be vaccinated [2]. Japan, which initially reported low rates of infection and death [3], now has the third-highest cumulative total of deaths in Asia [4]. Furthermore, more contagious variants are now emerging.

The COVID-19 pandemic has caused economic and work crises [5,6,7]. The first economic shock in Japan, which began in early 2020, was triggered by the initial outbreak of COVID-19 in China. Manufacturing in Japan stopped due to the closure of factories and restrictions on the logistics in China [5]. Border control measures were imposed to defend Japan against the entry of the virus, thus damaging the tourism industry, which depends heavily on Chinese tourists [8]. The second economic shock, which began in March, resulted from social distancing [9]. As a result of government and health authorities’ advice to refrain from unnecessary travel [3], people stayed at home to work and interacted online, thereby decreasing demand for products and services [7]. Although the government offered financial support at an early stage of the pandemic, focusing on employment and the operation of small and medium-sized enterprises, unemployment has continued to rise [10].

In addition, the pandemic has clearly placed strains on relationships [11,12], to which the government response has been insufficient [13]. To maintain social distancing, face-to-face interactions have been discouraged, and many cultural events, including the Tokyo Olympics, were cancelled or delayed [14]. Furthermore, stigma against infected people is a global concern [15,16]. In Japan, the government’s cluster-based approach, comprising an active epidemiological investigation and prompt measures taken in response to clusters of outbreaks, has resulted in stigma against certain social groups (e.g., the sex industry, health care workers, youth) [17]. Because the government has been unable to enforce lockdowns, they have instead been established as an informal standard [13]. As a result, rather than being legally punished for not adhering to social distancing, Japanese citizens have been subjected to physical and psychological mistreatment inflicted by family members, colleagues, and strangers, and, in extreme cases, to violence or slander [18].

These crises of health, economics, and social relationships may have severe implications for the mental health of workers [19]. The rate of telework use in Japan remains low [20], and workers face the risk of infection from clients and colleagues in the workplace [21,22]. Essential workers, such as health care workers, are also concerned about discrimination and slander during and after work [23]. In addition, the economic effects of COVID-19 have “trickle-down” implications for individual workers and their families [24]. Workers have been uncertain about their income and employment, and are confused by the sudden changes in their work environment, leading to a decline in mental health [25,26,27]. However, social support, which can help in stress management, has been restricted [28,29]. Since the pandemic, many studies have reported higher rates of anxiety, depression, post-traumatic stress disorder, psychological distress, and stress among both the general population [30,31,32] and health care workers [33]. These mental health effects can lead to job losses and suicide [34], and exacerbate economic harm [35]. In Japan in particular, socioeconomic crises are likely to increase the incidence of suicide [36,37,38]. In response to the economic crisis named the “Heisei great recession” during the 1990s, over 30,000 people committed suicide each year for 14 years [37].

It is important to recognize that COVID-19 may affect male and female workers unequally. Previous economic impacts have been closely related to increases in suicide rates among male workers [36,38], which may be related to a range of other job-related stressors, including job losses [24,36]. By comparison, women are more likely to have infection anxiety about COVID-19 [39], which increases the risk of depression and anxiety [31,33]. Moreover, Japanese women may be more vulnerable to economic shock because their average salaries are lower than those of men [40]. In the context of the COVID-19 pandemic, women increased their demand for household support, such as childcare when schools were closed and aged care for elderly parents. In addition, women were more likely to have to take on more housework and to lose their jobs than men [35,41]. Women are also more likely to follow social distancing policies [42], even though they prefer social support [43]. Furthermore, the number of suicides among Japanese women is reported to be increasing [35].

In line with the above concerns, this study examined workers’ psychological distress and difficulties in Japan during the COVID-19 pandemic. The experiences and lessons learned from past outbreaks, such as those of Zika and Ebola, show that robust gender analyses and informed gender-integrated measures are vital to strengthening access to and acceptability of mental health care [44]. In Japan, however, there are few large-scale epidemiological surveys of the working population. This study was designed to descriptively analyze the worker population’s current psychological distress and examine its relationship with demographic features. In addition, we conducted a detailed gender analysis of the stress environment by scoring the difficulties faced by workers with the item response theory (IRT) model [45].

## 2. Methods

This study was conducted in collaboration with the Fukushima Branch of the Japanese Trade Union Confederation and Fukushima Medical University.

### 2.1. Study Design and Participants

An online survey was conducted among workers in Fukushima Prefecture who belong to the Fukushima Branch of the Japanese Trade Union Confederation and related organizations. The questionnaire was created using a Google form and sent to the approximately 80,000 members through the branch mailing lists. Between 1 October 2020 and 23 November 2020, 3464 people responded.

During the survey period, the number of COVID-19-positive persons in Japan was 17,583 in October and 47,132 in November (there were 1765 cumulative deaths in October and 2138 in November) [46]. At the time, the national media noted that a third wave may have arrived [47]. In Fukushima Prefecture, 135 people were confirmed to be positive in October and 113 in November (6 cumulative deaths in both October and November) [48].

### 2.2. Measures

#### 2.2.1. Demographic Information

The survey collected demographic information on gender, age, nationality, family members, disability certification, underlying diseases, history of mental disorders, isolation experience, infection experience, industry type, job position, employment status, education attainment, and annual income (Table 1). Breakdowns of members of the Fukushima Branch of the trade union showed men, 69.0%; women, 31.0%; public employees, 36.8%; manufacturing workers, 33.4%; hotel and restaurant workers, 21.4%; transport workers, 6.2%; and health care workers, 2.2%. Therefore, the composition of the participant group represented that of the target population (we referred to an undisclosed name list for the breakdown values supplied by the manager of the Fukushima Branch).

#### 2.2.2. Psychological Distress

We assessed workers’ psychological distress during the COVID-19 pandemic in Japan. Distress was assessed with the Japanese version of the 6-item Kessler’s Psychological Distress Scale (K6) [49,50]. The K6 assesses feelings during the previous 30 days on a scale from 0 (none of the time) to 4 (all of the time). The validity of the Japanese version of the K6 has been confirmed [49,51].

#### 2.2.3. COVID-19-Related Stress and Effects

We measured the extent to which the pandemic affected participants’ mental state and lifestyle: we asked “How stressful has COVID-19 been for you to date?” and “How much has COVID-19 affected you to date?” on a scale from 1 (not at all) to 5 (very much).

#### 2.2.4. COVID-19-Related Difficulties

We used items related to difficulties from previous surveys in Japan [52,53,54]. The item set assessed notable difficulties related to COVID-19 during the survey (see Table 3).

### 2.3. Statistical Analyses

Participants with missing answers were excluded from the analysis. Four participants who selected “other” in the gender item were excluded because the sample size was too small to analyze. Finally, 3312 participants (95.6%) were included in the statistical analysis.

All analyses were performed in R v. 4.0.3 statistical software (R Core Team R: A Language and Environment for Statistical Computing; R Foundation for Statistical Computing: Vienna, Austria, 2020) [55], using the tools “stringr” [56] and “dplyr” [57] for setting the data frame; “stats” [55] for Pearson’s correlation, regression, confidence interval (CI), and deviance analyses; “performance” [58] for Nagelkerke’s *R*^2^ and variance inflation factor (VIF); “irtoys” [59] for IRT; “difR” [60] for detecting differential item functioning; and “polycor” [61] for polyserial correlation analyses. We considered results significant when *P* < 0.05.

#### 2.3.1. Japanese Workers’ Psychological Distress during the COVID-19 Pandemic

Psychological distress was assessed with the K6 score at two cut-off points: 5–9 points suggested psychological distress, and 10–24 points suggested mood and anxiety disorder [51,62]. The rate of K6 scores with ≥5 points was calculated by gender and industry type.

We conducted a stepwise multiple regression analysis of psychological distress in which the 14 sets of demographic data were entered as potential predictor variables. The stepwise analysis adopted a forward–backward stepwise selection method. To further analyze the effects of gender and industry type, we conducted a multiple regression analysis by adding a gender × industry type interaction term to the first model. In these analyses, the K6 score was entered without classifying by the cut-off points, and the Poisson regression model was applied. The model was evaluated against Nagelkerke’s *R*^2^ and analysis of deviance test. VIFs were 1.01–2.03, and we judged the impact of multicollinearity to be small.

#### 2.3.2. Assessment of COVID-19-Related Difficulties under Item Response Theory

To date, the pandemic has created a range of difficulties which are expected to diversify further. Previous studies have measured mental health impacts using Kessler’s Psychological Distress Scale [30,32], the Beck Depression Inventory [63], the Patient Health Questionnaire [64], the self-rating anxiety scale [65], the Depression, Anxiety and Stress Scale [66], and the specific Fear of COVID-19 scale [67]. These scales have a fixed set of items, are stable and straightforward to use, and allow for comparisons between surveys and for monitoring changes over time in specific concepts. However, scales with a fixed set of items are difficult to compare against past results when the content of the items is adjusted to a new situation. Because difficulties faced by workers vary by situation, we should consider how to scale the difficulties faced in the COVID-19 situation when analyzing them.

The IRT takes a person’s responses as a reflection of unobservable and latent traits [45]. It allows separate estimation of the parameters of the respondents’ trait values and item properties. Tests based on the IRT model do not need to use a fixed set of items. Thus, respondents’ scores can be compared even when there is a gap in the item sets among tests, such as measuring the difficulties faced by workers under the COVID-19 pandemic. In addition, separate estimation of respondents’ trait values and item properties allows examination of the characteristics of each difficulty faced by the workers without a dependency on the respondent groups. Therefore, we used the IRT model to score workers’ “COVID-19 related difficulties” (CORDI). The score on the CORDI test measures the overall severity of the difficulties and stressors that a person is facing under the COVID-19 pandemic.

We used a two-parameter logistic IRT model. We conducted marginal maximum likelihood estimation using the latent trait model for item parameters, and maximum likelihood estimation for participants’ trait parameters [45].

First, we explored the core assumptions of unidimensionality and local independence. The item set was collected to measure one trait (CORDI). The assumption of unidimensionality indicates that participants’ responses to the item set can be accounted for by the level of one factor. Therefore, items that are selected by too few or too many participants and that correlate lower with the total score should be excluded from the item set in the IRT model because they can impair the assumption of unidimensionality. We excluded items selected at a total rate of <0.1 or >0.9 [68]. We also excluded items with a Pearson’s correlation coefficient of <0.3 [68]. Then, we confirmed whether the item set was sufficiently unidimensional by drawing a scree plot. We interpreted the point at which the eigenvalue drops sharply in the plot as an appropriate number of factors.

Local independence assumes that item responses do not associate with each other when a dominant factor is controlled. This is a mathematical assumption used when estimating IRT parameters. If this assumption is not fulfilled, estimates of the respondents’ traits are inaccurate. We identified local independence with Yen’s Q3 statistic [69]. Q3 values, which are coefficients of the residual correlation matrix between items, were calculated from expected scores from estimated IRT parameters. Using 0.2 as the criterion, combinations of items with suspicious assumptions of local independence were integrated [70].

Some of the items have the potential to respond differently between men and women, even if they face the same degree of difficulties related to the COVID-19 pandemic. These items are said to have *differential item functioning* [71]. We separately estimated the item parameters for men and women, and then equated the parameters between men and women to detect differential item functioning. Because their separately estimated item parameters may have different distributions of the CORDI scores, we ensured they were comparable by equating their distributions [71]. We used the mean-sigma method for adjusting results with reference to men. We compared item parameters between men and women by conducting Lord’s chi-squared test [72] to detect differential item functioning.

Participants’ CORDI scores were estimated from the final item parameters. We separately estimated the rate of selection of each item at a CORDI score of 0 in men and women. We calculated the test information function by summing the item information functions to confirm the reliability of the item [59], and identified the maximum values of the test information function and their CORDI scores in men and women. We also confirmed the convergent validity of the CORDI score by polyserial correlation analysis with the scores of COVID-19-related stressors and effects.

#### 2.3.3. Relationships between CORDI and Other Characteristics

We analyzed the relationship between CORDI score (by gender and industry type) and participants’ characteristics. We also conducted a stepwise multiple regression analysis of the CORDI score in which the 14 demographic variables were entered as potential predictors. The stepwise analysis used a forward–backward stepwise selection method. To further analyze effects of gender and industry type, we conducted a multiple regression analysis by adding a gender × industry type interaction term to the model in the stepwise analysis. VIFs were 1.03–1.31, and the impact of multicollinearity was judged to be small.

In addition, we analyzed correlations between the CORDI score and the K6 score.

## 3. Results

### 3.1. Japanese Workers’ Psychological Distress under the COVID-19 Pandemic

K6 showed that 30.9% of participants had suspected psychological distress (scores of 5–9) and 19.8% had suspected mood and anxiety disorders (scores of 10–24). They comprised 455 men (46.9% of 970 men) and 190 women (59.0% of 322 women) among public employees (*n* = 645, 49.9% of total); 473 men (51.5%) and 91 women (54.5%) among manufacturing workers (*n* = 564, 51.9%); 219 men (46.0%) and 156 women (58.0%) among hotel and restaurant workers (*n* = 375, 50.3%); 50 men (43.9%) and 14 women (73.9%) among transport workers (*n* = 64, 48.1%); and 11 men (55.0%) and 21 women (60.0%) among health care workers (*n* = 32, 58.1%).

Stepwise regression analysis for psychological condition (K6 score) suggested 10 predictive variables, of which eight were significant (Table 2). K6 scores were significantly higher for women, age, underlying diseases, history of mental disorder, infection experience, and higher income, and significantly lower for hotel and restaurant workers, transport workers, and administrative workers.

Multiple regression analysis with the addition of gender × industry type interaction showed the model to be statistically significant (Nagelkerke’s *R*^2^ = 0.18, χ^2^(24) = 668.08, *P* < 0.05; data not shown). With reference to male public employees, K6 scores were higher among female public employees (*b* = 0.13, 95% CI = [0.08, 0.18], *z* = 4.75, *P* < 0.05), male manufacturing workers (*b* = 0.05, 95% CI = [0.01, 0.09], *z* = 2.44, *P* < 0.05), and female transport workers (*b* = 0.34, 95% CI = [0.14, 0.53], *z* = 3.42, *P* < 0.05), and lower among male transport workers (*b* = −0.24, 95% CI = [−0.33, −0.14], *z* = −4.96, *P* < 0.05).

### 3.2. Assessment of COVID-19-Related Difficulties under Item Response Theory

#### 3.2.1. Assumption of Unidimensionality

**Figure 1:**
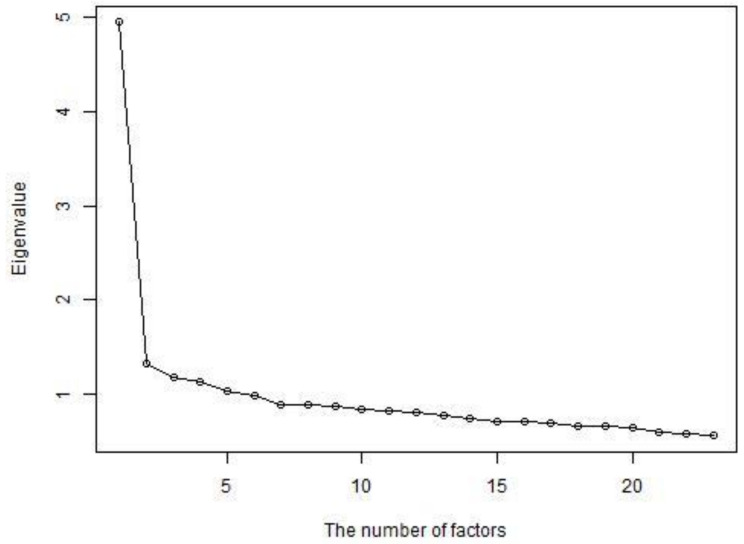
Scree plot of the 29 items of the COVID-19-related difficulties (CORDI) item set.

Items were selected at rates of 0.02 to 0.72. Pearson’s *r* between each item score and the total score ranged from 0.19 to 0.57. Ten items that few participants selected (<0.1) or that had a low correlation with total score (*r* < 0.03) were excluded: “1 Concerns about being laid off or unemployed”, “3 Government compensation is difficult to get”, “9 Workload has increased”, “14 No one to ask for help when I need it”, “16 PCR test is not available for me”, “18 Housing has been lost or is under threat of being lost”, “22 Family conflicts have increased”, “26 Food supplies have or may become insufficient”, “32 Increased burden of housework”, and “36 I have too much time”. Eigenvalues in the scree plot decreased rapidly from 1 to 2 factors and very little thereafter (Figure 1). Therefore, the item set without these 10 items was taken to show unidimensionality.

#### 3.2.2. Assumption of Local Independence

We integrated the following combinations of items (CoI) because their Q3 values were >0.2: CoI 1, “2 Income has decreased or is under threat of decreasing” and “4 The business performance of my company has deteriorated or is under threat of deteriorating”; CoI 2, “11 Anxiety that I may be infected” and “12 Anxiety that my family members may be infected”; CoI 3, “19 My family’s or my personal information and activities were made public or are under threat of being made public if we are infected”, “20 In the COVID-19 context, discrimination in public spaces was experienced or anticipated”, and “21 In the COVID-19 context, slander by neighbours and co-workers was experienced or anticipated”; CoI 4, “27 Daily necessities have or may become insufficient”, and “28 Preventive products have or may become insufficient”; and CoI 5, “30 Child’s school or kindergarten has been closed or restricted” and “37 Adverse effects on child development worry me”. Table 3 shows the items we used and explanations of the final item set for IRT.

#### 3.2.3. Differential Item Functioning between Men and Women

With reference to men, the equating coefficient of discrimination was 0.67 and that of difficulty was 0.26. Figure S1 shows the relationship between CORDI scores and the estimated response rate of each item, based on the item parameters after adjustment.

Differential item functioning was significant (*P* < 0.05) in 16 items (Table 4): “5 Worry that the Japanese economy will get worse”, “6 Work styles have changed”, “7 Teleworking is not possible or is difficult”, “10 Work efficiency has decreased”, “13 Fatigue from taking measures against COVID-19 infection”, “23 Difficulty in interacting and getting along with people”, “24 Uncertain about what is the correct information about SARS CoV-2”, “25 Every day, news about SARS CoV-2 appears on the Internet and TV”, “29 Living expenses have increased”, “31 Reduced opportunities to interact with friends and distant family members”, “34 Lack of exercise for myself and my family”, “38 No longer able to go out as much as before”, and “39 Worry that I will not be able to live if I am infected”, and three combined items: CoI 1, CoI 2, and CoI 4.

#### 3.2.4. Reliability and Convergent Validity of the Item Set

Items estimated at a higher rate (> 30%) at a CORDI score of 0 were CoI 2 (infection: men, 85.8%; women, 82.9%), item 38 (restricted movement: men, 47.1%; women, 59.6%), item 31 (reduced social interaction: men, 47.8%; women, 54.6%), item 5 (worsening Japanese economy: men, 54.5%; women, 40.2%), CoI 3 (public identification and shunning: men, 37.4%; women, 38.7%), and item 25 (news: men, 35.9%; women, 30.6%). Items estimated at a higher rate in men were CoI 1 (lost income: men, 49.7%; women, 28.4%) and item 23 (difficulty in interacting with people: men, 32.6%; women, 25.1%).

**Figure 2:**
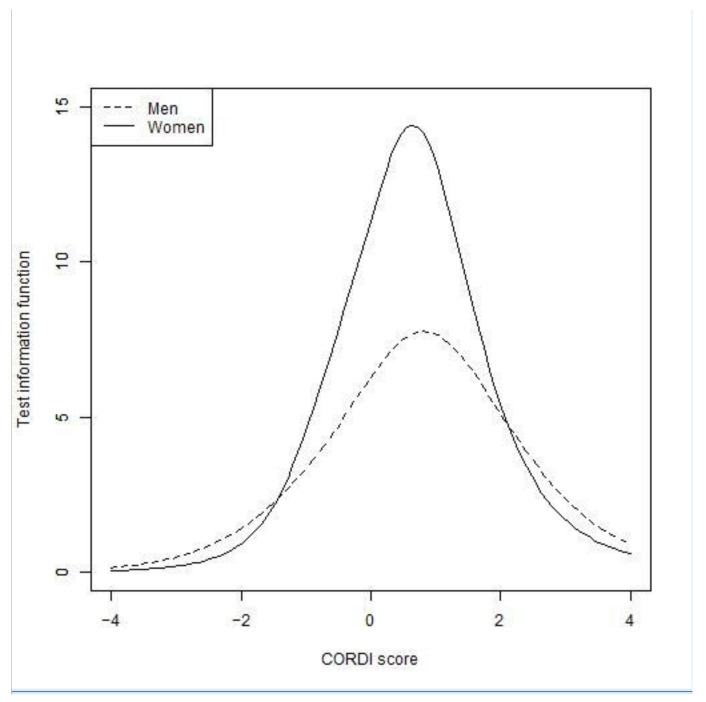
Test information functions of the CORDI item set of men and women.

The maximum test information function in men was 7.75 at a CORDI score of 0.80 and that in women was 14.40 at a CORDI score of 0.64 (Figure 2).

The polyserial correlation coefficient with COVID-19-related stress was 0.47 (*P* < 0.05) and that with the COVID-19-related effect was 0.41 (*P* < 0.05), confirming the convergent validity of the CORDI score.

### 3.3. Relationships between CORDI and Other Variables

The mean CORDI score was −0.23 (±1.40 SD) in men and 0.14 (±0.95) in women among public employees; −0.07 (±1.37) in men and 0.17 (±1.15) in women among manufacturing workers; −0.07 (±1.35) in men and 0.23 (±1.00) in women among hotel and restaurant workers; 0.14 (±1.06) in men and 0.30 (±1.21) in women among transport workers; and 0.08 (±0.83) in men and 0.22 (±0.95) in women among health care workers.

Stepwise analysis suggested six significant (*P* < 0.05) predictive variables (Table 5). CORDI scores were significantly higher for women, age, family members, underlying diseases, manufacturing workers, and hotel and restaurant workers, and lower for part-time workers.

Multiple regression analysis with the addition of gender × industry type interaction showed the model to be statistically significant (*R*_adj_^2^ = 0.05, *F*(13, 3298) = 14.32, *P* < 0.05). With reference to male public employees, higher CORDI scores were found among female public employees (*b* = 0.51, 95% CI = [0.34, 0.67], *t* = 6.13, *P* < 0.05), male manufacturing workers (*b* = 0.17, 95% CI = [0.05, 0.28], *t* = 2.89, *P* < 0.05), and male transport workers (*b* = 0.25, 95% CI = [0.01, 0.50], *t* = 2.02, *P* < 0.05).

The polyserial correlation coefficient with the K6 score was 0.22 (*P* < 0.05).

## 4. Discussion

### 4.1. Psychological Distress among Japanese Workers

We investigated the effects of COVID-19 on the mental health of Japanese workers and their stressors. Workers’ K6 scores were significantly worse (30.9% with scores of 5–9, 19.8% with scores of 10–24) than in 2019 (17.6%, 9.7%) [62].

Women and past and current medical conditions, including COVID-19 infection and psychiatric diseases, were associated with high psychological distress levels. In contrast, unlike in previous studies [31], low income was negatively associated with psychological distress. Because many of the participants (93.2) were full-time workers, more low-income participants are needed to compare with the average population in Japan, where the proportion of part-time workers is nearly 30% [10].

We developed the CORDI test to analyze the difficulties faced by workers in Japan. Its convergent validity was confirmed by the moderate correlations of the item scores of COVID-19-related stress and effect. The CORDI score correlated with the K6 score (Section 3.3), and the test highlighted some difficulties caused by COVID-19 and gender gaps in these difficulties.

As suggested by the CORDI test, infection anxiety, perceived economic insecurity, and reduced social interaction posed the greatest difficulty (lowest item difficulty scores), indicating that workers generally faced these difficulties during the pandemic. Mental health is severely affected by infection anxiety [31,33], economic insecurity [24,36], and reduced social interaction [12,28]. Our findings show that these stressors may be basic issues that should take priority over other stressors in the COVID-19 era. In Japan, measures have been taken in regard to the health and economic crises, but there is a lack of measures to address the decline in social interaction [13]. Policymakers should offer more support for and health education about adequate social interaction during the pandemic to support workers’ mental health, especially among women (refer to [11,15,73,74,75]), while avoiding conflicts with health measures [76]. Note, however, that the stronger the effect of COVID-19 becomes, the more stressors there are to be treated. At the time of this survey, Japanese workers were also concerned about stigma (refer to [15,16]) and information overload (refer to [77,78]).

### 4.2. Gender Gap in Difficulties Caused by the COVID-19 Pandemic

Women had higher K6 and CORDI scores than men. This result suggests that the pandemic has had a greater effect on women, as reported previously [31,33]. Furthermore, the CORDI test suggested gender differences in some difficulties faced by workers, possibly reflective of traditional gender differences. The item parameters of the CORDI test suggested three types of gaps:

The first type is the lower item difficulty scores (greater difficulty) among women than among men. More women perceived lack of exercise, increasing cost of living, anxiety about inadequate supplies of daily necessities, and anxiety about infection prevention tools as stressors than men. The restrictions on outdoor exercise and the need to stay at home have reduced physical exercise during the pandemic [79]. Because people know that daily exercise reduces lifestyle diseases [80], lack of exercise may cause health anxiety. In Japan, although the rate of daily exercise is higher among men [80], this result may indicate that women were more concerned about, not only their own health, but also that of their families. Moreover, lack of money and supplies was a greater stressor for women than for men. Previous studies noted that financial stress has a strong effect on men’s mental health [36,38]. However, our results may indicate that women are in charge of managing household finances and procuring supplies in Japan.

The second type is the higher item discrimination scores among women than among men: the rate for men is slightly higher when the CORDI score is low, but the rate for women is much higher when the CORDI score is high. Women were more sensitive to stress at a lower intensity of effects through the decreased social interaction, restrictions on outings, and information overload. Women are more likely to seek social support than men for depression and anxiety [81,82], and may be more likely to suffer from stress due to information overload, because they are more engaged in collecting information to mitigate their anxiety [83]. In addition, lack of telework, fatigue due to disease avoidance, confusion about information, and uncertainty about living after infection increased strongly when the effect of COVID-19 was higher. In such a situation, employers should try to create a female-friendly telework system, and to relieve the anxiety that leads to compulsive preventive behavior and information gathering that we observed among participants.

The third type concerns difficulties that men are more likely to face, notably economic insecurity and changes in working style. These results may reflect the traditional gender roles by which men go to work and women stay at home to do housework, even though most of the men and women in this study were full-time workers [37]. Men’s financial stress may reflect the employers’ situation, whereas women’s financial stress may reflect the household situation. To adapt to the rapid changes in working styles, male workers may need support and training in changes in working style [20,84]. Surprisingly, men were more likely to be stressed by infection anxiety than women, even though previous studies have reported the opposite [31,33]. This result could indicate that the item properties of the CORDI test were estimated separately from the respondent’s trait values by the IRT model; that is, although women are more prone to infection anxiety because they are more affected by COVID-19 overall, men may be more likely to be assessed as having infection anxiety if both men and women are affected to the same extent.

### 4.3. Gender Differences by Industry Types

The results suggest some gender differences among industry types. Manufacturing workers perceived a stronger effect of the pandemic, particularly men, who perceived it more intensely and were more stressed. The initial outbreak in China economically affected manufacturers during the early stages of the pandemic [5]. Our results among male manufacturing workers suggest that that the effect of this industry type had an economic bias. Similarly, male transport workers were strongly affected, although their psychological stress was not high.

Psychological distress was higher among women in public employees and transport workers. Many public employees, such as health care workers, respond early to COVID-19, thus, this result is consistent with the psychological consequences experienced by hospital nurses [33]. Public workers may perceive themselves as being more susceptible to mass surveillance regarding adherence to public health strategies. Strategies, such as not going out, may be more stressful for women [81,82], thus explaining the gender differences in public employees. Moreover, the results observed in female transport workers may reflect the predominance of men in this industry; if men represent the majority in an organization, material and social support may favor them.

### 4.4. Limitations

This study has several limitations. The first is the low response rate of only 3464 responses from approximately 80,000 workers canvassed; although the distribution of respondents’ attributes did not differ markedly from that of the target population, we need to be careful about its representativeness. In addition, because the sample size of health care workers in this study was small (*n* = 55), we did not find statistically significant differences in psychological stress among them compared to other industry types. However, this result does not indicate that health care workers have similar psychological distress to workers in other industries.

Furthermore, our findings do not cover all the characteristics of the Japanese worker population. Members of the Japanese Trade Union Confederation generally have relatively stable employment. A previous study showed that the problems of female workers are more serious in part-time employment [35]. Our results, in contrast, show that the effects are serious among women in both full-time and part-time employment, but further research is needed on the difficulties specific to part-time workers.

Finally, this study comprised a cross-sectional survey, but the COVID-19 situation is constantly changing. The CORDI test allows the effects of COVID-19 in the same dimension to be scored even when the content of the difficulties changes. Future studies should use this advantage to investigate the longitudinal changes in the effects of gender and industry type over time.

## 5. Conclusions

We revealed that Japanese workers face severe psychological distress, a variety of difficulties, and gender gaps during the COVID-19 pandemic. Coping with infection anxiety, economic insecurity, and reduced social interaction may be fundamental to protecting workers affected by the pandemic. However, it should not be overlooked that the development of a vaccine will resolve only one of these stressors. Additionally, support for workers tends to focus on job-related difficulties, such as economic insecurity and anxiety about changes in the work environment. A lack of support for non-job-related difficulties, such as reduced social interaction and life insecurity, may allow the mental health of female workers to decline. Therefore, gender-integrated supports should be designed to ensure that male and female workers have equitable access to, and benefits from, mental health care.

## Figures and Tables

**Table 1:** Demographic information.

		*n*	%
All		3464	
Gender	men	2613	75.4%
	women	847	24.5%
	other	4	0.1%
Age	10s	43	1.2%
	20s	744	21.5%
	30s	981	28.3%
	40s	1036	29.9%
	50s	597	17.2%
	60s	61	1.8%
Nationality	Japanese	3445	99.5%
	other	18	0.5%
Family members	presence	1694	48.9%
	absence	1769	51.1%
Disability certificate	presence	36	1.0%
	absence	3426	98.9%
Underlying diseases	presence	281	8.1%
	absence	3179	91.8%
History of mental disorders	presence	114	3.3%
	absence	3347	96.6%
Isolation experience	experienced	51	1.5%
	not experienced	3411	98.5%
Infection experience	experienced	35	1.0%
	not experienced	3428	99.0%
Industry type	public employees	1350	39.0%
	manufacturing workers	1135	32.8%
	hotel and restaurant workers	788	22.7%
	transport workers	135	3.9%
	health care workers	55	1.6%
Job position	administrative position	883	25.5%
	not administrative position	2581	74.5%
Employment status	full-time	3230	93.2%
	not full-time	233	6.7%
Education attainment	junior high/high school	1760	50.8%
	vocational or junior college	493	14.2%
	university or graduate school	1211	35.0%
Annual income	<150	177	5.1%
[×10^4^ JPY]	150–199	98	2.8%
	200–299	335	9.7%
	300–399	615	17.8%
	400–499	747	21.6%
	500–699	1027	29.6%
	700–999	422	12.2%
	≥1000	42	1.2%

**Table 2:** Stepwise Poisson regression analysis of K6 score.

Predictor		*b*	95% CI
Gender (ref = men)		0.10 *	[0.06, 0.14]
	women		
Age		0.03 *	[0.01, 0.04]
Nationality (ref = others)		−0.16	[−0.33, 0.02]
	Japanese		
Underlying diseases (ref = absence)		0.15 *	[0.10, 0.20]
	presence		
History of mental disorder (ref = absence)		0.63 *	[0.56, 0.69]
	presence		
Infection experience (ref = absence)		0.21 *	[0.07, 0.34]
	presence		
Industry types (ref = public employees)			
	manufacturing workers	0.03	[−0.002, 0.07]
	hotel and restaurant workers	−0.06 *	[−0.10, −0.02]
	transport workers	−0.17 *	[−0.26, −0.09]
	health care workers	0.01	[−0.10, 0.12]
Job positions (ref = none)		−0.04 *	[−0.08, −0.0002]
	administrative worker		
Education attainment (ref = junior high/ high school)			
	vocational or junior college	−0.02	[−0.05, 0.0004]
	university or graduate school	−0.01	[−0.05, 0.02]
Annual income (ref = <150 [ten thousand yen])			
	150–199	−0.24 *	[−0.35, −0.14]
	200–299	−0.07	[−0.16, 0.01]
	300–399	0.13 *	[0.06, 0.21]
	400–499	0.07 *	[0.01, 0.14]
	500–699	0.02	[−0.04, 0.07]
	700–999	0.07 *	[0.03, 0.12]
	≥1000	0.01	[−0.02, 0.05]
Nagelkerke’s *R*^2^		0.18	
Test of deviance (χ^2^(20))		648.53 *	

*P < 0.05; 95% CI, 95% confidence interval.

**Table 3:** Items of COVID-19-related difficulties (CORDI) in this survey.

	*n*	%	Final Item Set for IRT
1 Concerns about being laid off or unemployed	304	9.2%	
2 Income has decreased or is under threat of decreasing	1071	32.3%	integrated (CoI 1)
3 Government compensation is difficult to get (e.g., strict restrictions, complexity of procedures)	210	6.3%	
4 The business performance of my company has deteriorated or is under threat of deteriorating	1076	32.5%	integrated (CoI 1)
5 Worry that the Japanese economy will get worse	1737	52.4%	included
6 Work styles have changed (e.g., telework, staggered work hours)	764	23.1%	included
7 Teleworking is not possible or is difficult	1028	31.0%	included
8 Criteria for work absence when symptoms similar to those of COVID-19 appear (low fever, lassitude etc.) are unclear	659	19.9%	included
9 Workload has increased	694	21.0%	
10 Work efficiency has decreased	712	21.5%	included
11 Anxiety that I may be infected	2370	71.6%	integrated (CoI 2)
12 Anxiety that my family members may be infected	2379	71.8%	integrated (CoI 2)
13 Fatigue from taking measures against COVID-19 infection (e.g., hand washing, gargling, masks)	1007	30.4%	included
14 No one to ask for help when I need it	327	9.9%	
15 Concerns that hospitals and other facilities will be unable to cope with the increased number of infected people	970	29.3%	included
16 PCR test is not available for me	203	6.1%	
17 Unable to go to the hospital owing to chronic illness or cold	627	18.9%	included
18 Housing has been lost or is under threat of being lost	50	1.5%	
19 My family’s or my personal information and activities were made public or are under threat of being made public if we are infected	1147	34.6%	integrated (CoI 3)
20 In the COVID-19 context, discrimination in public spaces was experienced or anticipated (e.g., restricted work attendance, forced changes in work content)	785	23.7%	integrated (CoI 3)
21 In the COVID-19 context, slander by neighbours or co-workers was experienced or anticipated	890	26.9%	integrated (CoI 3)
22 Family conflicts have increased (including domestic violence)	96	2.9%	
23 Difficulty in interacting and getting along with people (e.g., having to wear a mask when talking to people)	1253	37.8%	included
24 Uncertain about what is the correct information about SARS CoV-2	944	28.5%	included
25 Every day, news about SARS CoV-2 appears on the Internet and TV	1347	40.7%	included
26 Food supplies have or may become insufficient	188	5.7%	
27 Daily necessities have or may become insufficient (e.g., tissue paper, toilet paper, diapers, sanitary products, preserved foods)	412	12.4%	integrated (CoI 4)
28 Preventive products have or may become insufficient (e.g., masks, disinfectant)	808	24.4%	integrated (CoI 4)
29 Living expenses have increased (e.g., food expenses)	613	18.5%	included
30 Child’s school or kindergarten has been closed or restricted	457	13.8%	integrated (CoI 5)
31 Reduced opportunities to interact with friends and distant family members	1727	52.1%	included
32 Increased burden of housework (e.g., cooking, laundry, cleaning, childcare)	297	9.0%	
33 No longer able to attend weddings, funerals, school or company events	1085	32.8%	included
34 Lack of exercise for myself and my family	627	18.9%	included
35 Lost/limited transportation due to avoidance of crowds	538	16.2%	included
36 I have too much time	323	9.8%	
37 Adverse effects on child development worry me (e.g., lost learning, decreased academic performance, lack of exercise)	640	19.3%	integrated (CoI 5)
38 No longer able to go out as much as before (e.g., hobbies, lessons, shopping, eating out, live concerts)	1746	52.7%	included
39 Worry that I will not be able to live if I am infected (e.g., securing food, taking care of children)	961	29.0%	included

**Table 4:** Gender differences of item parameters.

Item No.	Men		Women		Lord’s χ^2^
	Discrimination	Difficulty	Discrimination	Difficulty	
5	1.23 (0.07)	−0.15 (0.04)	1.24 (0.15)	0.32 (0.07)	51.50 *
6	0.93 (0.07)	1.38 (0.09)	0.72 (0.17)	2.60 (0.51)	17.00 *
7	1.04 (0.07)	1.02 (0.07)	1.55 (0.17)	0.71 (0.07)	7.61 *
8	1.02 (0.07)	1.68 (0.11)	0.92 (0.16)	1.76 (0.24)	0.57
10	0.89 (0.07)	1.51 (0.11)	0.93 (0.18)	2.36 (0.38)	55.21*
13	0.92 (0.06)	1.11 (0.08)	1.41 (0.17)	0.85 (0.08)	7.36 *
15	1.76 (0.10)	0.81 (0.04)	2.13 (0.21)	0.73 (0.05)	3.76
17	1.22 (0.08)	1.57 (0.09)	1.49 (0.19)	1.27 (0.11)	1.75
23	1.33 (0.08)	0.55 (0.05)	2.19 (0.21)	0.50 (0.05)	17.24 *
24	1.26 (0.08)	1.00 (0.06)	2.14 (0.22)	0.75 (0.06)	13.92 *
25	1.33 (0.08)	0.43 (0.04)	1.93 (0.19)	0.42 (0.05)	11.72 *
29	1.16 (0.08)	1.69 (0.10)	1.82 (0.21)	1.10 (0.08)	13.78 *
31	1.30 (0.07)	0.07 (0.04)	1.92 (0.20)	−0.10 (0.05)	11.94 *
33	1.36 (0.08)	0.77 (0.05)	1.63 (0.18)	0.68 (0.06)	2.74
34	1.05 (0.08)	1.71 (0.11)	1.88 (0.21)	1.16 (0.09)	14.14 *
35	1.35 (0.09)	1.62 (0.09)	1.84 (0.22)	1.29 (0.10)	3.93
38	1.44 (0.08)	0.08 (0.04)	1.89 (0.20)	−0.21 (0.06)	18.65 *
39	1.79 (0.10)	0.84 (0.04)	2.67 (0.26)	0.64 (0.05)	9.34 *
CoI 1	0.72 (0.05)	0.02 (0.06)	0.91 (0.14)	1.02 (0.13)	114.99 *
CoI 2	1.47 (0.09)	−1.22 (0.06)	2.57 (0.29)	−0.62 (0.07)	54.52 *
CoI 3	1.23 (0.07)	0.42 (0.04)	1.43 (0.16)	0.32 (0.06)	1.13
CoI 4	1.18 (0.08)	1.32 (0.08)	2.05 (0.20)	0.63 (0.05)	41.25 *
CoI 5	1.27 (0.08)	1.23 (0.07)	1.17 (0.16)	1.26 (0.13)	1.03

Item numbers as in Table 3. Women’s parameters after equating: (SE); * *P* < 0.05.

**Table 5:** Stepwise multiple regression analysis for CORDI score.

Predictor	*b*	95% CI
Gender (ref = men) women	0.45 *	[0.33, 0.56]
Age	0.15 *	[0.10, 0.19]
Family members (ref = none) presence	0.28 *	[0.19, 0.38]
Underlying diseases (ref = absence) presence	0.19 *	[0.03, 0.35]
Industry types (ref = public employees)		
manufacturing workers	0.13 *	[0.03, 0.23]
hotel and restaurant workers	0.14 *	[0.02, 0.26]
transport workers	0.22	[−0.01, 0.44]
health care workers	0.14	[−0.20, 0.48]
Employment status (ref = full-time) not	−0.21 *	[−0.41, −0.01]
*R*_adj_^2^	0.05	
*F* (9, 3302)	20.36 *	

*P < 0.05; 95% CI, 95% confidence interval.

## Data Availability

Not applicable.

## Supplementary Materials

The following are available online at https://www.mdpi.com/article/10.3390/ijerph18168656/s1, Figure S1: the item characteristic curves of male and female workers for each item.
