# Supplementary material for: Traditional Gender Differences Create Gaps in the Effect of COVID-19 on Psychological Distress of Japanese Workers
Source: Int J Environ Res Public Health. 2021 Aug 16;18(16):8656. doi: 10.3390/ijerph18168656 (PMC8391574; doi:10.3390/ijerph18168656)
Supplement: Supplementary file 1 [file ijerph-18-08656-s001.zip › ijerph-1313543-supplementary.pdf]

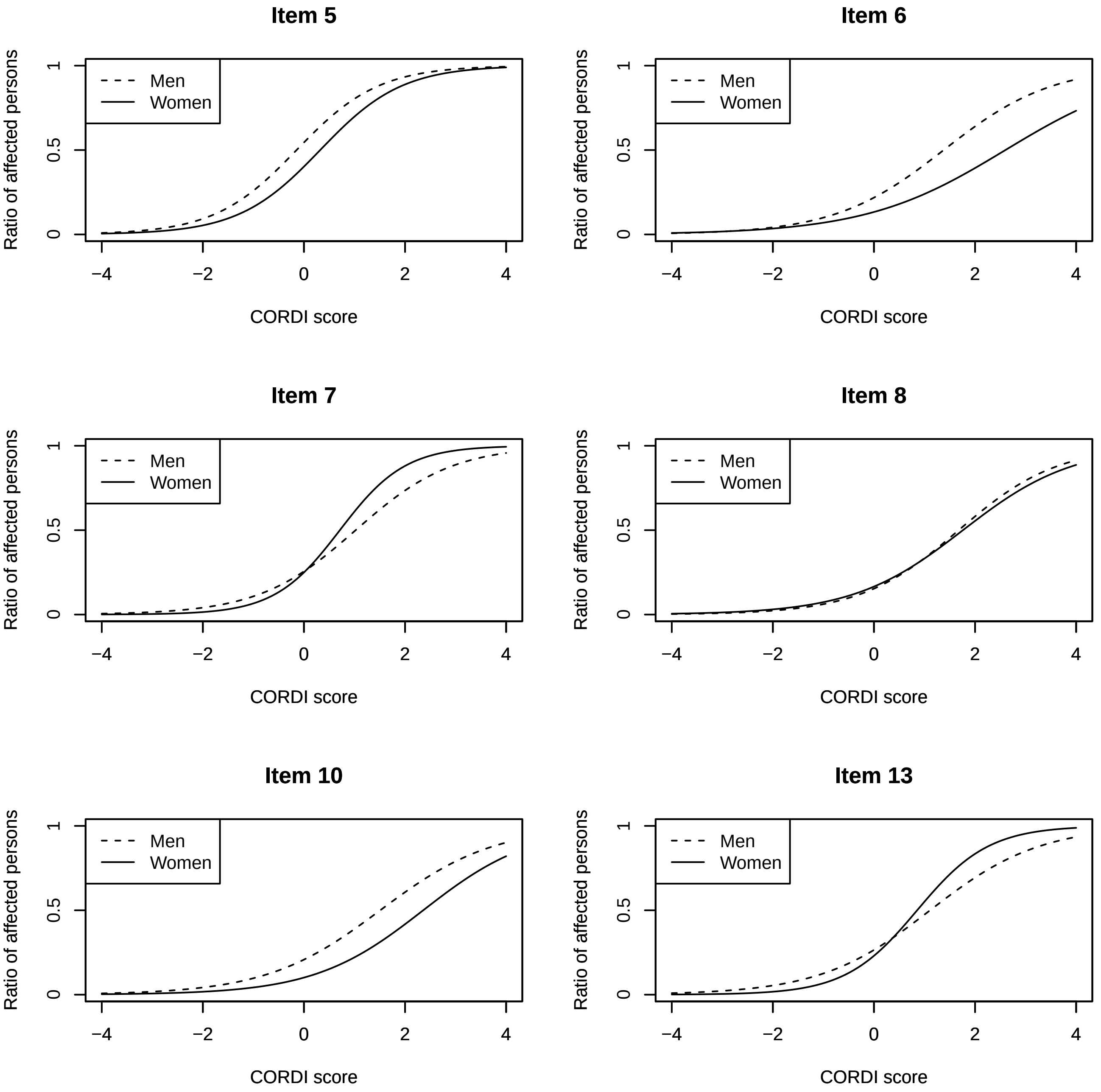
Item 5
Item 6
Item 7
Item 8
Item 10
Item 13
Men
Women
Ratio of affected persons
CORDI score
0
0.5
1
−4
−2
0
2
4

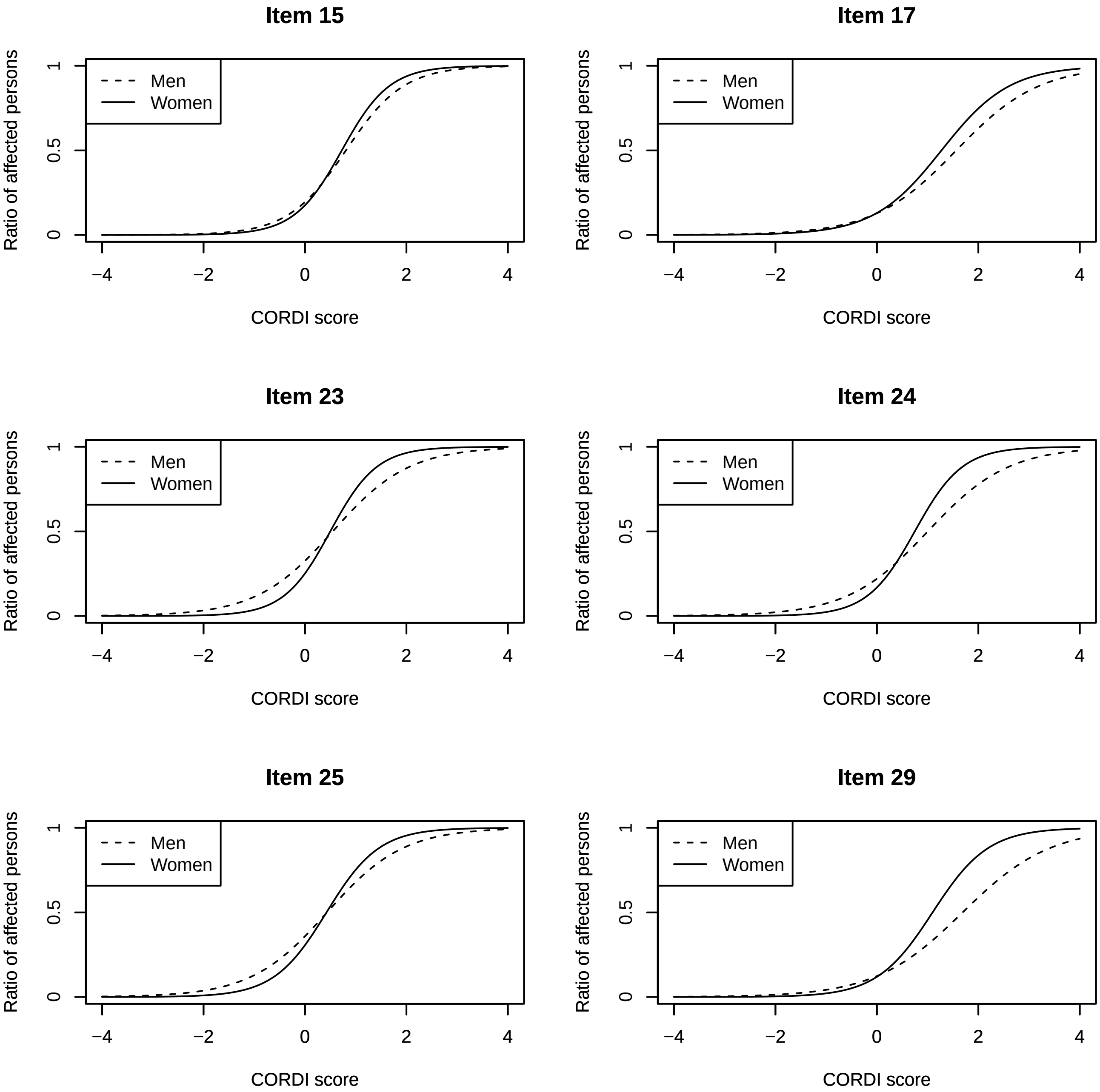
Item 15
Item 17
Item 23
Item 24
Item 25
Item 29
Men
Women
Ratio of affected persons
CORDI score
0
0.5
1
−4
−2
0
2
4

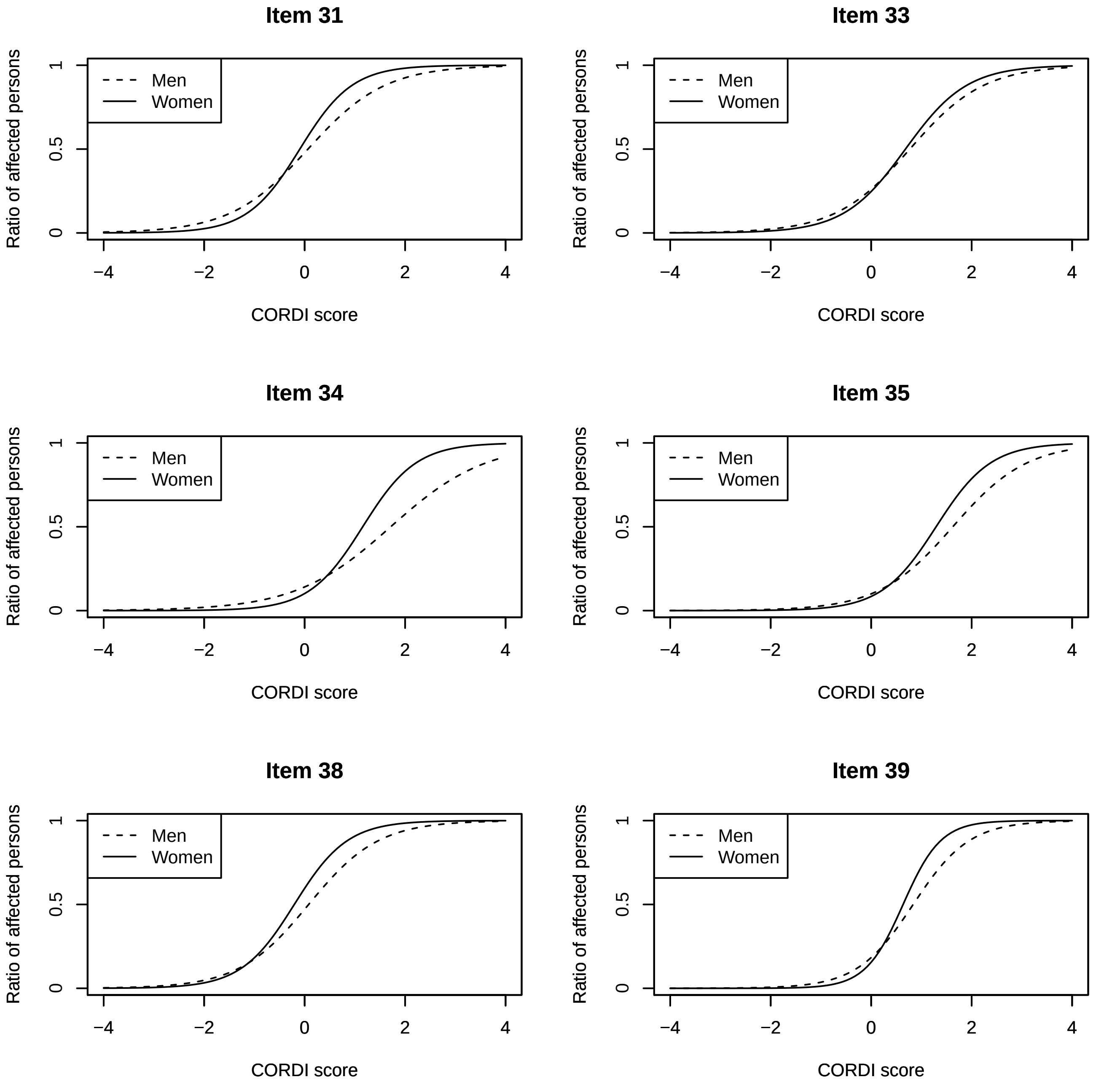
Item 31
Men
Women
Ratio of affected persons
0
0.5
1
−4
−2
0
2
4
CORDI score
Item 33
Men
Women
Ratio of affected persons
0
0.5
1
−4
−2
0
2
4
CORDI score
Item 34
Men
Women
Ratio of affected persons
0
0.5
1
−4
−2
0
2
4
CORDI score
Item 35
Men
Women
Ratio of affected persons
0
0.5
1
−4
−2
0
2
4
CORDI score
Item 38
Men
Women
Ratio of affected persons
0
0.5
1
−4
−2
0
2
4
CORDI score
Item 39
Men
Women
Ratio of affected persons
0
0.5
1
−4
−2
0
2
4
CORDI score

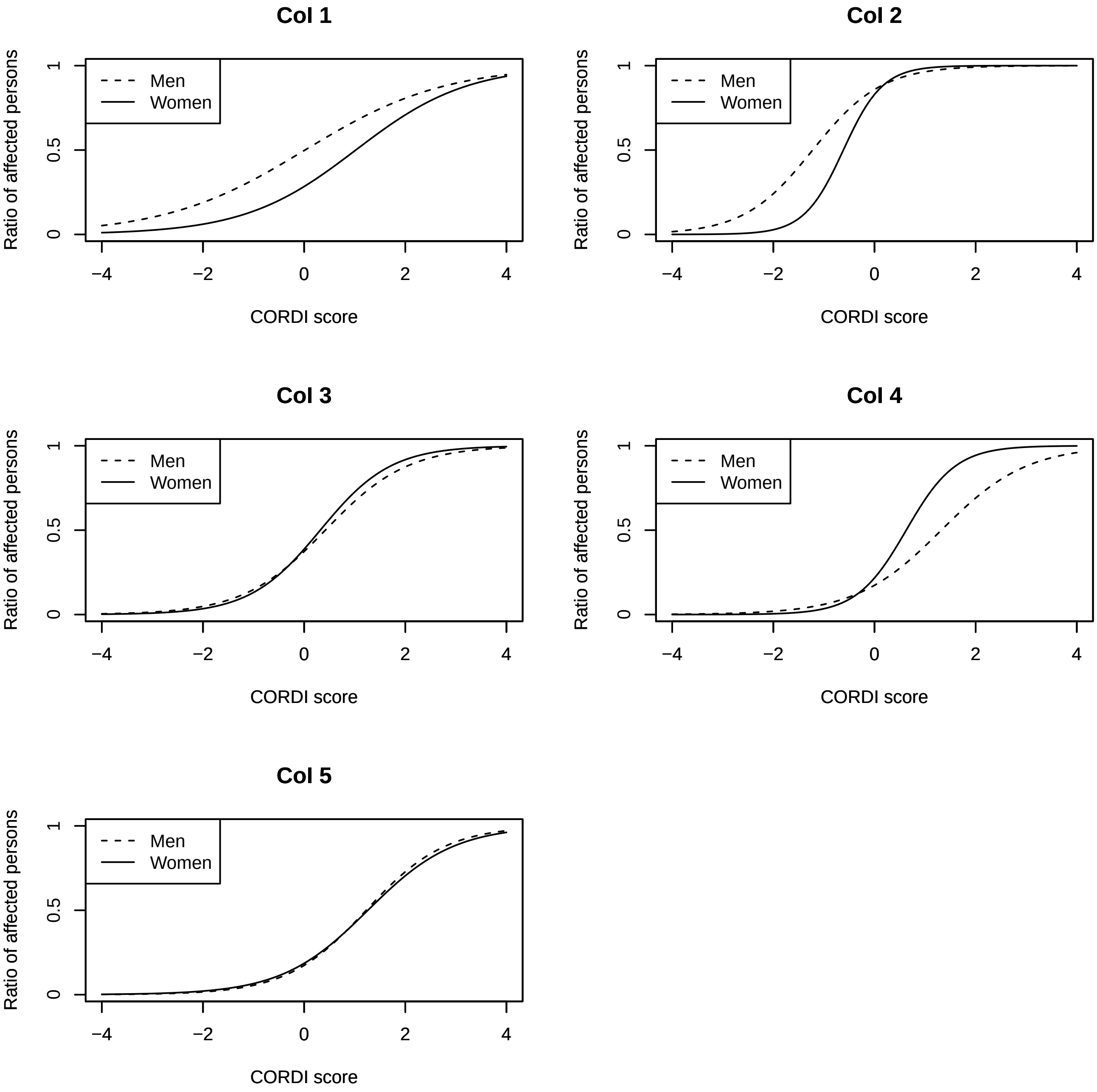

Col 1
Men
Women
Ratio of affected persons
0
0.5
1
−4
−2
0
2
4
CORDI score
Col 2
Men
Women
Ratio of affected persons
0
0.5
1
−4
−2
0
2
4
CORDI score
Col 3
Men
Women
Ratio of affected persons
0
0.5
1
−4
−2
0
2
4
CORDI score
Col 4
Men
Women
Ratio of affected persons
0
0.5
1
−4
−2
0
2
4
CORDI score
Col 5
Men
Women
Ratio of affected persons
0
0.5
1
−4
−2
0
2
4
CORDI score
